# Resection of the Gastric Tube Reconstructed through the Retrosternal Route without Sternotomy

**DOI:** 10.1155/2017/5862871

**Published:** 2017-02-19

**Authors:** Masahiro Kimura, Yasuyuki Shibata, Kotaro Mizuno, Hironori Tanaka, Motoki Hato, Satoshi Taniwaki, Yoichiro Mori, Nobuo Ochi, Takaya Nagasaki, Shuhei Ueno, Yuki Eguchi

**Affiliations:** Department of Surgery, Nagoya City East Medical Center, 2-23 Wakamizu 1, Chikusa-ku, Nagoya 464-8547, Japan

## Abstract

With advances of combined modality therapy, prognoses in esophageal cancer have been improving. After resection of esophageal cancer, the development of gastric tube cancer is a risk. While such cancer in an early stage can be cured endoscopically, total gastric tube resection is indicated in advanced stages. A 68-year-old man underwent subtotal esophagectomy reconstructed with a gastric tube through the retrosternal route. Gastric cancer was found one and a half years postoperatively. The gastric tube was resected without sternotomy. This is the first report of a patient undergoing resection of the gastric tube reconstructed through the retrosternal route without sternotomy.

## 1. Introduction

There are three routes for reconstruction of a gastric tube after esophagectomy, namely, subcutaneous, retrosternal, and posterior mediastinal. In the case of a subcutaneous route, removal of the gastric tube is relatively straightforward. In the case of resection of conduits reconstructed via the two other routes, surgical resection is more demanding. In the case of the posterior mediastinal route, resection of the gastric tube is extremely difficult because of adhesions with the lung and mediastinum. In the case of the retrosternal route, a median sternotomy has traditionally been necessary for resection. The median sternotomy is routinely performed in cardiac surgery and for mediastinal tumor resection. This procedure has the risk of serious complications, including mediastinitis or sternal dehiscence. We herein present our new technique to resect the gastric tube reconstructed through the retrosternal route without the need for sternotomy, which we hypothesized would decrease postoperative pain as well as the rate of complications by reducing the surgical stress involved with a sternotomy.

## 2. Case Report

A 68-year-old man was admitted to our department with dysphagia. An upper endoscopic examination revealed a midthoracic esophageal cancer and an early gastric cancer of the antrum. Considering the progress of esophageal and gastric cancer, we conducted subtotal esophagectomy and partial gastrectomy. The gastric cancer was resected as the gastric tube was formed, and sufficient margin was secured. The gastric tube was lifted to the neck via a retrosternal route. Histopathological analysis showed moderately differentiated squamous cell carcinoma of the esophagus invading the adventitia without lymph node metastasis (pT3N0M0: stage II) [1] and signet-ring cell carcinoma which involved the mucosal layers of the stomach (pT1aN0M0: stage Ia) [2]. One and a half years postoperatively with R0 resection, he underwent surveillance upper endoscopic examination. There was a mass on the lesser curvature of the distal gastric tube, different from the site of the previous cancer, which was biopsied. Histology revealed a poorly differentiated adenocarcinoma. The depth of invasion reached the submucosal layer, and the lesion was directly above the staple line. The lesion was too difficult to treat endoscopically, and lymph node metastases were suspected.

## 3. Operation

**Figure 1:**
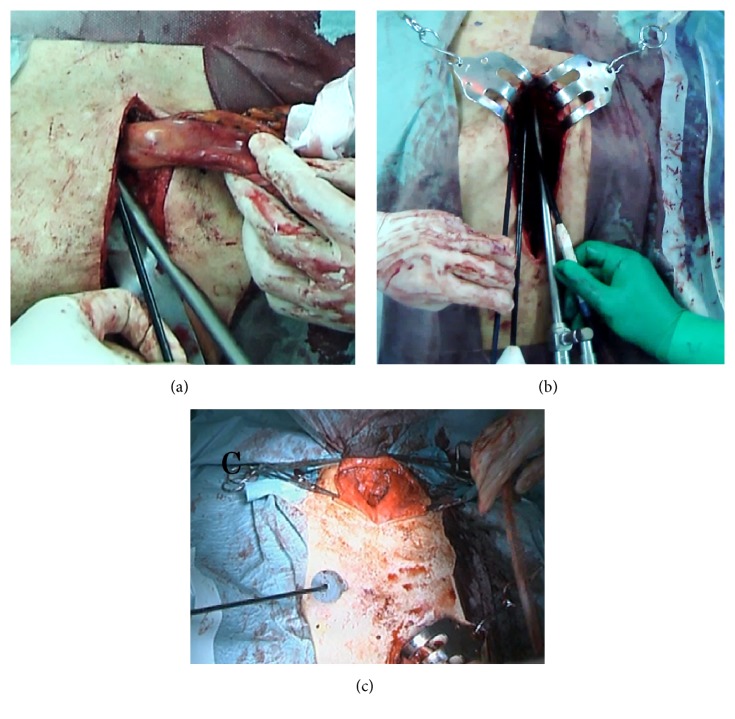
(a) Distal part of the gastric tube. (b) Endoscopic view of the dissection from the ventral side. (c) Neck incision and right chest thoracoscopic port.

Prior to resection of the gastric tube, the right gastroepiploic and gastric artery lymph nodes, along with the suprapyloric and infrapyloric lymph nodes, were dissected. By ligating the blood flow first, operative bleeding during resection of the gastric tube could be reduced. The duodenum was then transected about 1 cm distal to the pyloric ring with an automatic stapling device. Seromuscular sutures were placed on the stump of the duodenum (Figure 1(a)).

**Figure 2:**
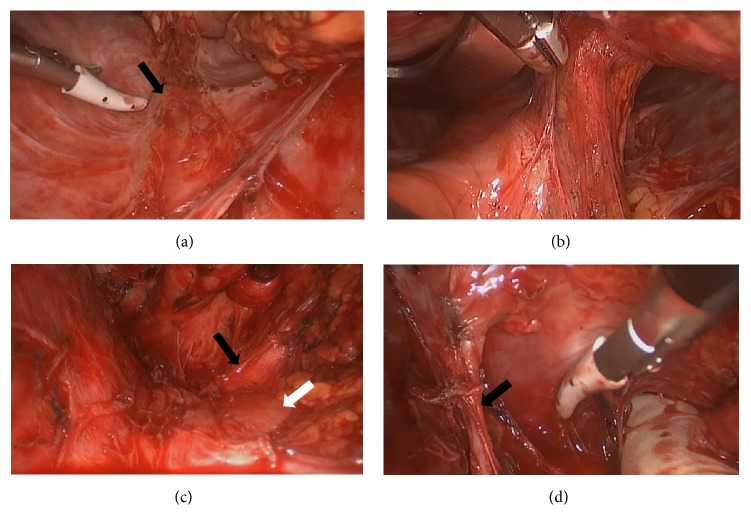
(a) Adhesion between the pericardium and the staple line of the gastric tube (black arrow). (b) Adhesion between the back surface of the sternum and the omentum. (c) Superior vena cava (black arrow) and left brachiocephalic vein (white arrow). (d) Right internal mammary vein (black arrow).

Initially, the gastric tube was dissected from the surrounding tissue under direct vision. After about 10 cm of dissection, both costal arches were elevated to expand the space of the mediastinum. The adhesions were most dense on the anterior and posterior surfaces of the mediastinum (Figure 2(a)). The back of the sternum could be adherent to the omentum or stomach wall anteriorly. The pericardium could be adherent to the staple line of the gastric tube posteriorly (Figure 2(b)). However, these surfaces were relatively safe to dissect, as they could be done so with an ultrasonic coagulation-cutting device while observing the dissection endoscopically to ensure no intraluminal violation (Figure 1(b)). Posterior adhesions were relatively loose and could be taken down bluntly in many cases.

While the gastric tube was still adherent to the sternum, the gastric tube could be dissected off the pericardium. If the gastric tube was first dissected anteriorly, the gastric tube fell posteriorly and visualization for the posterior dissection became difficult. As the dissection advanced in the cephalad direction, the visual field became narrow and the operation became difficult. Therefore, when approximately two-thirds of the dissection was complete, a thoracoscopic port was inserted in the right chest in an intercostal space (Figure 1(c)). By inserting forceps from this port, retraction of the gastric tube was facilitated. At this point, a skin incision was made in the neck. The cervical esophagus was identified and separated from the surrounding tissue. The amount of dissection done in the neck was limited as the visual field is small.

In this operation, damage to large blood vessels must be avoided. The superior vena cava, brachiocephalic vein, and internal mammary vein were all located in the upper mediastinum (Figure 2(c)). After identification of the inferior vena cava, dissection must proceed being mindful to not violate this vessel. It was also critical to be mindful of the internal mammary vein branching off from brachiocephalic vein (Figure 2(d)). However, after the identification and dissection of these large vessels, the dissection of the gastric tube closer to the head was relatively straightforward. Once the gastric tube was completely dissected, the cervical esophagus was divided near the anastomosis and the gastric tube was removed. It took 315 minutes to remove the gastric tube.

A drainage tube was inserted into the retrosternal space. The ileum and colon were used for reconstruction in a subcutaneous route. End-to-side anastomosis with esophagus and terminal ileum was performed by hand sewing. Histopathological analysis showed signet-ring cell carcinoma which involved the submucosal layers of the stomach (pT1aN0M0: stage Ia). R0 operation was completed. Postoperative pain relief was in the form of patient-controlled analgesia. The patient was discharged 2 weeks after the operation without complications. He is followed up with no additional treatment.

## 4. Discussion

As recent advances in the diagnosis and treatment of esophageal cancer improve patient survival after esophagectomy, a second primary cancer involving the gastric tube is encountered with higher frequency. Postoperative endoscopic examination, therefore, is imperative and may be useful for the early detection [3–7]. With the early detection of gastric tube cancer, endoscopic mucosal resection can be curative and the stomach can be preserved [8].

On the other hand, total gastric tube resection may be indicated for advanced gastric tube cancer. The stomach is the organ most commonly used as an esophageal replacement because of the simplicity of the operative technique and the rich blood supply. However, the reconstruction route is dependent on the stage of the cancer and the condition of the patient. Although the resection of a gastric tube in a subcutaneous position is straightforward, this route is rarely selected. On the other hand, resection of the gastric tube in a retrosternal or posterior mediastinal position is difficult and requires a more invasive surgery due to adhesions with surrounding organs and vessels. It is traditionally necessary to open the chest again in the case of a conduit in the posterior mediastinal position. In the case of a retrosternal position, a median sternotomy may be necessary.

The median sternotomy is used widely in cardiac and mediastinal surgery. However, postoperative complications after this approach, including mediastinitis, are severe and sometimes life-threatening. With the development of endoscopic surgery, resection of mediastinal tumors can be done thoracoscopically without sternotomy. However, no reports exist describing gastric tube resection without sternotomy.

There are two possible ways to reach the gastric tube without sternotomy. One is via the pleural cavity and the other is via the mediastinum. We selected the latter for the following three reasons:

- Most of the adhesions exist in the direction of the body axis and dissection is easy.
- The risk of lung damage is minimized.
- We can convert to a sternotomy if any bleeding is encountered from large vessels without changing the patient position.

**Figure 3:**
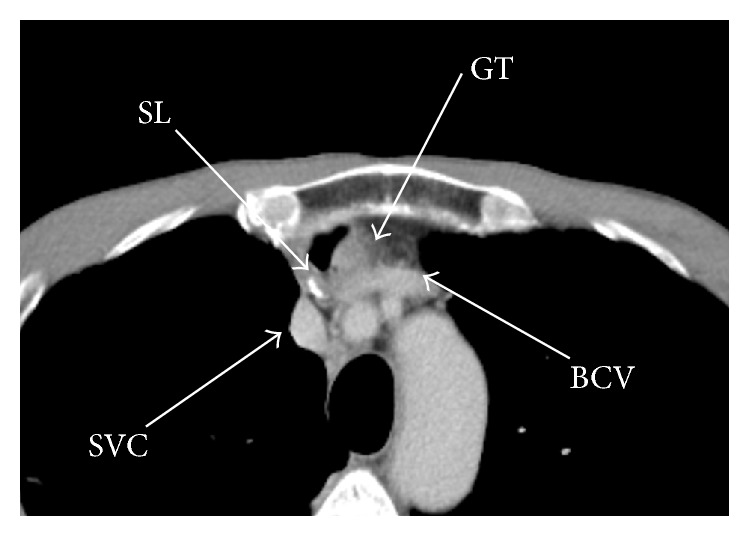
Computed tomography of the anterior mediastinum. GT: gastric tube, SL: staple line, SV: superior vena cava, and BCV: brachiocephalic vein.

The most important aspect of our technique is the working space. As the dissection proceeds towards the head, the working space becomes narrower and the large vessels appear (Figure 3). In the initial surgery, the left brachiocephalic vein is dissected off the sternum, and the gastric tube is placed between the sternum and left brachiocephalic vein. Thus, dissection of adhesions in this area must be done with great care, with additional attention to not cause inadvertent injury to the internal thoracic vein. It is important to note the positional relationship between the blood vessels and the gastric tube preoperatively by computed tomography. It is not advisable to begin the dissection from the cephalad portion of the gastric tube. It is occasionally useful to insert a port through an intercostal space to facilitate dissection in the upper side of the mediastinum. In this case, the port was inserted through the right fourth intercostal space.

In the midline, only the sternum lies in front of the gastric tube. In the conventional method, the sternum is transected after the dissecting the gastric tube from the sternum. After the gastric tube is resected, reconstruction can be performed using the small intestine or colon. However, this procedure is complicated and the complication rate including anastomotic leak is high. Mediastinitis and sternal dehiscence can also occur, with high morbidity and mortality.

Using our technique, the gastric tube can be resected with an expanded view using an endoscope without the need for a sternotomy. This technique can also reduce the risk of serious infection and postoperative pain and should be considered in the resection of a gastric tube reconstructed through the retrosternal route.
